# Incidentally Discovered Cerebellar Ganglioglioma in an Adult Following a Motor Vehicle Accident: A Case Report and Literature Review

**DOI:** 10.7759/cureus.109132

**Published:** 2026-05-18

**Authors:** Oludamilola Adeshina, Shane Musick, Nicolas Phan

**Affiliations:** 1 Neurological Surgery, Marshall University Joan C. Edwards School of Medicine, Huntington, USA; 2 Neurology, Marshall University Joan C. Edwards School of Medicine, Huntington, USA

**Keywords:** brain tumors cns tumors, cerebellum, ganglioglioma, incidentaloma, neurosurgery

## Abstract

Gangliogliomas (GGs) are rare central nervous system neoplasms composed of neuronal and glial cells. This report details a rare case of a 32-year-old male patient with an incidentally discovered cerebellar GG following a motor vehicle accident. The literature review highlights the variability in symptoms, age, and imaging findings, emphasizing the importance of considering GG in differential diagnoses for infratentorial tumors. The patient's treatment involved subtotal resection due to the risk of significant neurological deficits, with ongoing monitoring for recurrence. This case demonstrates a generally favorable prognosis of cerebellar GGs when appropriately managed surgically.

## Introduction

Gangliogliomas (GGs) are rare primary central nervous system (CNS) neoplasms composed of neoplastic glial and dysplastic neuronal elements. They account for less than 1% of intracranial tumors and are more commonly diagnosed in younger patients, with approximately 80% presenting before 30 years of age [1,2]. According to the 2021 World Health Organization Classification of Tumors of the Central Nervous System, ganglioglioma is classified among glioneuronal and neuronal tumors and is designated CNS WHO grade 1 [3].

GGs most commonly arise supratentorially, above the tentorium cerebelli, particularly in the temporal lobe, where they frequently present with long-standing epilepsy or seizure-related symptoms [2,4]. Infratentorial tumors arise below the tentorium and include posterior fossa lesions involving the cerebellum, brainstem, or fourth ventricle. Cerebellar and posterior fossa GGs are rare, with a limited number of cases reported [5-11]. Symptoms vary by size and location and may include headache, nausea, vomiting, gait disturbance, ataxia, cranial nerve deficits, or increased intracranial pressure [5,6,10,11]. Incidentally discovered cerebellar GGs are uncommon because most reported patients present after a prolonged symptomatic course [2,4].

Radiological findings of GGs are highly variable and may include cystic components, calcifications, contrast enhancement, susceptibility signal, or minimal surrounding edema, contributing to diagnostic uncertainty; diagnostic evaluation begins with magnetic resonance imaging (MRI), although findings may overlap with other posterior fossa lesions [4,6,7,11]. Definitive diagnosis requires histopathology showing mixed glial and neuronal elements, commonly with dysplastic ganglion cells and astrocytic proliferation [6-8]. Immunohistochemical markers such as GFAP, synaptophysin, and Ki-67, along with molecular markers including BRAF V600E and isocitrate dehydrogenase (IDH) status, may provide additional diagnostic information [12,13]. The increased use of cross-sectional imaging in trauma and other screening contexts has led to more frequent detection of asymptomatic intracranial lesions, often referred to clinically as “incidentalomas.” In this setting, distinguishing benign, low-grade lesions from tumors requiring urgent intervention can be challenging.

Surgical resection remains the primary diagnostic and therapeutic modality and is generally associated with favorable outcomes [1,5-7,11,13,14]. After complete resection of low-grade ganglioglioma, management is typically centered on surveillance rather than routine adjuvant therapy [1]. Herein, we report an incidentally discovered cerebellar GG in a 32-year-old adult following a motor vehicle accident (MVA), notable for adult presentation, absence of preoperative neurological symptoms, midline inferior vermian location, subtotal resection, and stable follow-up.

## Case presentation

A 32-year-old male patient was transferred to our facility following a MVA. The patient was an unrestrained driver traveling at approximately 45 miles per hour when he struck a telephone pole due to distracted driving. Upon arrival at the scene of the accident, Emergency Medical Services noted airbag deployment, orthopedic fractures to the right leg, and a Glasgow Coma Scale rating of 15. The patient had no mention of head or neck pain or loss of consciousness and was neurologically intact. 

Upon arrival at the Emergency Department, he underwent a routine non-contrast head CT scan, which demonstrated an incidental midline cerebellar mass with calcifications, measuring approximately 2.2 × 2.3 cm, without hydrocephalus or acute intracranial hemorrhage (Figure 1). He had no focal neurological deficits attributable to the lesion. The patient had no documented prior neurological symptoms, relevant family history of CNS tumors, or significant past neurological history. Cranial nerves were intact, strength and sensation were preserved, and no dysmetria, ataxia, or gait abnormality was documented. Two days after the accident, a brain MRI with and without contrast was performed using sequences including susceptibility-weighted angiography (SWAN), T1-weighted, T2-weighted, fluid-attenuated inversion recovery (FLAIR), and diffusion-weighted imaging/apparent diffusion coefficient (DWI/ADC).

**Figure 1:**
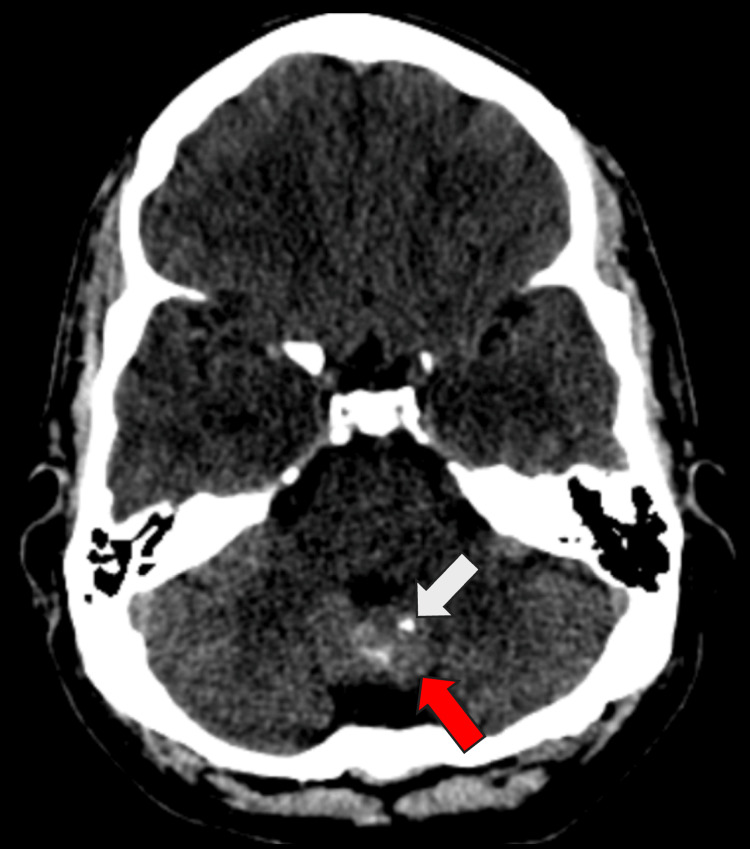
Axial CT of head showing midline cerebellar mass with calcifications. The white arrow indicates a hyperdense calcified focus within the lesion; the red arrow indicates the midline cerebellar mass.

Imaging demonstrated an enhancing midline inferior cerebellar mass measuring 2.5 × 2.1 × 2.5 cm, without significant mass effect, surrounding edema, or hydrocephalus (Figure 2). Because the CT and MRI were different imaging modalities, the slight difference in reported measurements was not interpreted as definite interval growth. Rather, the lesion was felt to be incidental and did not require urgent neurosurgical intervention, and close outpatient follow-up was recommended after recovery from his traumatic injuries.

**Figure 2:**
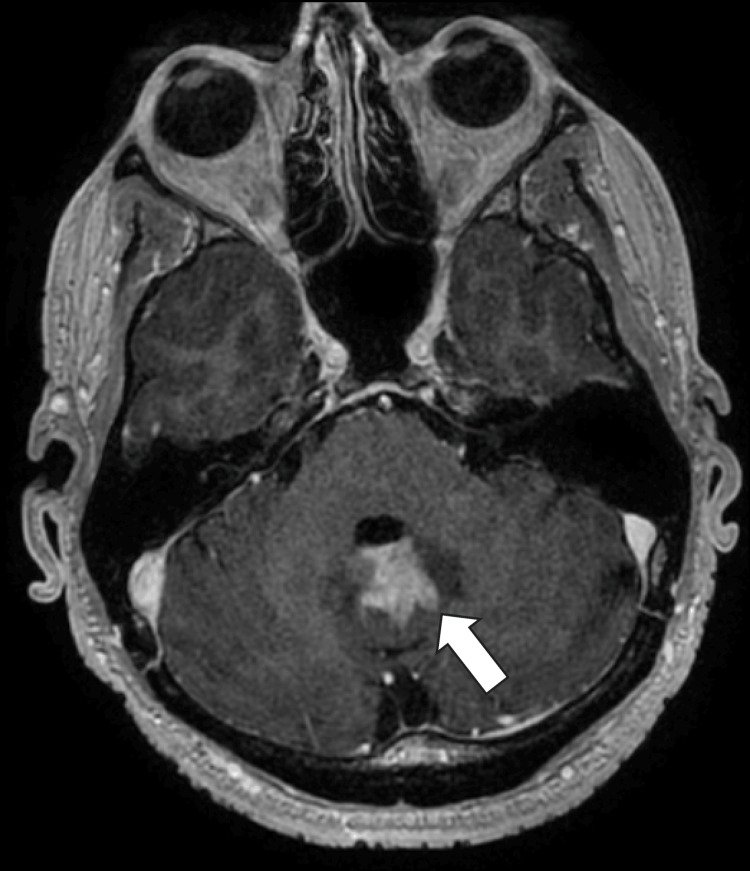
Post-contrast axial T1-weighted FLAIR MRI demonstrating mass in the midline of the inferior cerebellum The arrow indicates a mass in the midline cerebellum measured 2.5 × 2.1 × 2.5 cm. FLAIR, fluid-attenuated inversion recovery.

At the six-week follow-up, the patient reported headaches but remained neurologically intact. Repeat brain full MRI protocol demonstrated mild interval growth in the lesion’s craniocaudal dimension, measuring 2.7 cm compared with 2.5 cm previously, with slight partial compression of the fourth ventricle but no hydrocephalus (Figure 3).

**Figure 3:**
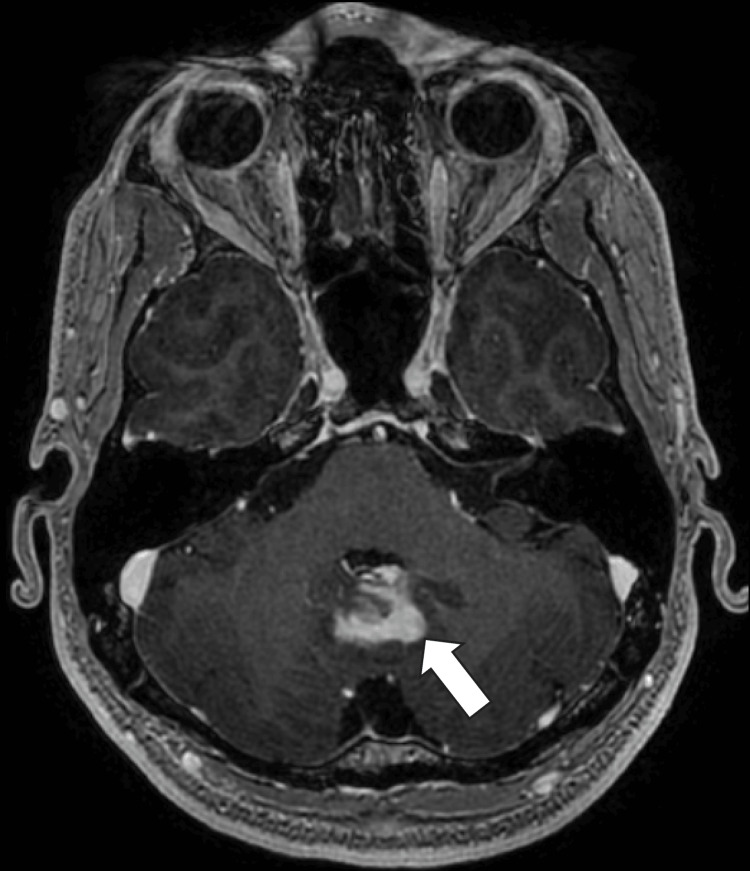
Repeat post-contrast axial T1-weighted FLAIR MRI showing increased tumor size with slight partial compression of the fourth ventricle. The arrow indicates increased tumor size at six-week follow-up, demonstrating mild interval craniocaudal growth to 2.7 cm. FLAIR, fluid-attenuated inversion recovery.

The lesion remained enhancing and infiltrative in appearance, with an internal susceptibility signal on the SWAN sequence corresponding at least in part to the punctate calcifications seen on CT; blood products could not be excluded (Figure 4). Given the enhancing lesion, internal susceptibility signal, and calcifications, CT angiography (CTA) was obtained to exclude an underlying vascular lesion such as a cavernous malformation or arteriovenous malformation; CTA demonstrated no vascular abnormality and redemonstrated peripheral punctate calcifications. No surrounding edema or restricted diffusion was identified. The differential diagnosis included low-grade glial or glioneuronal tumor, pilocytic astrocytoma, ependymoma, hemangioblastoma, metastasis, and inflammatory lesion. Given the interval growth and indeterminate imaging features, surgery was recommended for tissue diagnosis and maximal safe resection. The patient expressed understanding of the incidental diagnosis and the rationale for subtotal resection.

**Figure 4:**
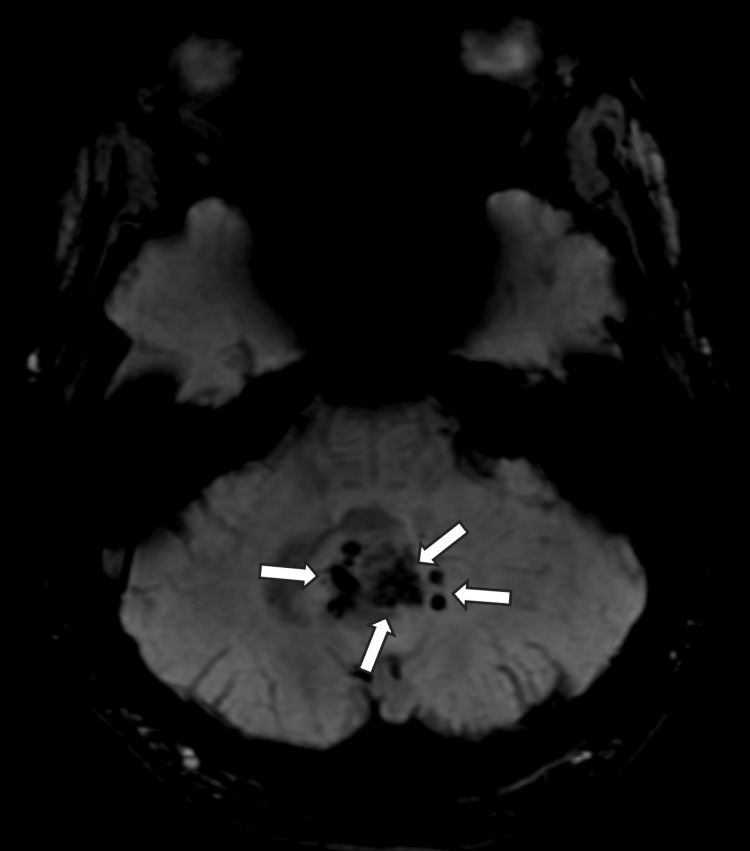
Evidence of blood products on axial susceptibility-weighted angiography (SWAN) MRI. The arrows indicate tumor-related products susceptible to blood or calcification.

One month later, preoperative laboratory evaluation, including complete blood count, basic metabolic panel, and coagulation studies, was within normal limits, with no thrombocytopenia or coagulopathy identified. The patient underwent midline suboccipital craniotomy with removal of the posterior lip of the foramen magnum using stereotactic guidance and microscopic magnification. The lesion was localized to the midline inferior vermis between the cerebellar tonsils. A midline inferior vermian approach was used, and the tumor was resected piecemeal. During anterior dissection, the tonsils were elevated, and the fourth ventricle was identified as an anatomic landmark. Because the lesion appeared infiltrative and extended superiorly into the vermis, gross total resection was not considered safely feasible. The decision to stop at subtotal resection was based primarily on the lesion’s infiltrative superior vermian extension and proximity to critical posterior fossa structures, including the roof of the fourth ventricle, rather than on navigation or frozen section findings alone. Frozen section showed a hypercellular lesion, but could not distinguish neoplastic from inflammatory etiologies. The surgery lasted approximately 2.5 hours.

Postoperatively, the patient was extubated and transferred to the intensive care unit in stable condition for neurological monitoring. He remained neurologically intact, with nausea as his only reported immediate postoperative symptom. His postoperative incisional pain was managed with oral oxycodone and intravenous hydromorphone. The nausea resolved spontaneously by the following morning. The patient was discharged the next day with education on wound management, pain medication, antiemetics, and follow-up appointments.

Postoperative day 1 CT demonstrated resection of the central tumor component with residual lateral calcification in the inferior cerebellum, concerning for residual tumor. Frozen section demonstrated a hypercellular lesion, but was limited in distinguishing a neoplastic process from an inflammatory lesion intraoperatively. On permanent sections, histopathological examination demonstrated dystrophic calcifications, perivascular lymphoid infiltrates, glial cell proliferation, and dysplastic neurons on hematoxylin and eosin staining, supporting a final diagnosis of ganglioglioma, CNS WHO grade 1 (Figure 5). Pathological examination was performed at an external facility, and immunohistochemical and molecular diagnostic testing, including GFAP, synaptophysin, NeuN, CD34, Ki-67, BRAF V600E, and IDH status, was not performed. The diagnosis was rendered based on permanent histopathological sections demonstrating mixed glial and neuronal elements with dysplastic neurons, glial cell proliferation, dystrophic calcifications, and perivascular lymphoid infiltrates.

**Figure 5:**
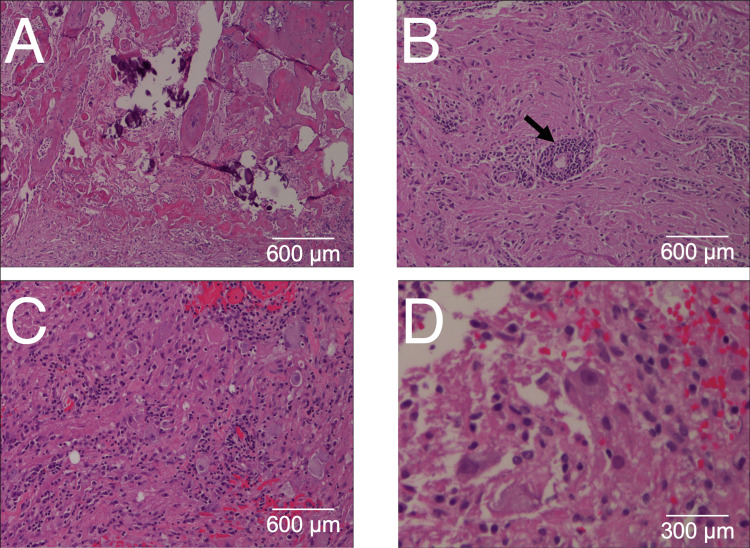
Histopathological hematoxylin and eosin (H&E) staining. A: Dystrophic calcification within the tumor matrix, corresponding to calcifications identified on preoperative CT imaging, 200x, scale bar = 600 μm. B: Perivascular lymphoid inflammation within a fibrillary glial background (black arrow), 200x, scale bar = 600 μm. C: Mixed glioneuronal tumor morphology with scattered dysplastic neuronal elements, 200x, scale bar = 600 μm. D: Dysplastic neurons: large cells with prominent nucleoli, binucleation, abnormal clustering, 400x, scale bar = 300 μm.

At two-month follow-up, MRI showed stable postoperative changes with residual tumor at the midline and superior resection margin, without hydrocephalus (Figure 6).

**Figure 6:**
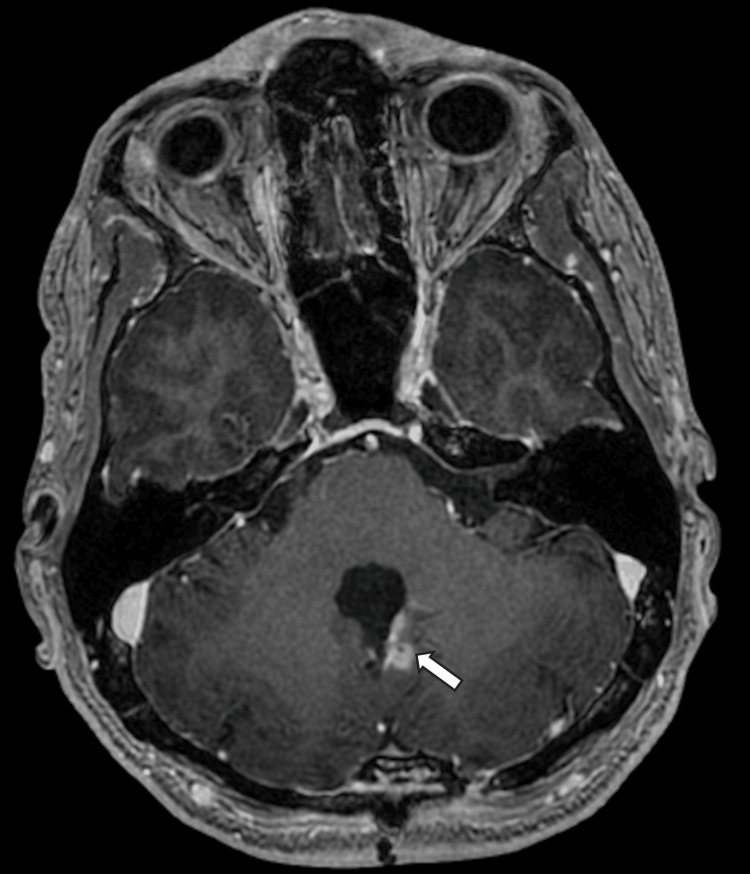
Repeat post-contrast axial T1-weighted FLAIR MRI demonstrating residual tumor at resection margin. The arrow indicates postoperative MRI, demonstrating residual tumor along the midline and superior resection margin without interval progression. FLAIR, fluid-attenuated inversion recovery.

He mentioned a return of his nausea with morning emesis and was prescribed 4 milligrams of oral ondansetron to take every six hours. At five-month follow-up, the patient corresponded to a modified Rankin Scale score of 1 due to mild daily headaches not interfering with daily activities, being treated with over-the-counter analgesics. His nausea has improved, and imaging was stable. Given the low-grade histology, absence of neurological deficits, and stable postoperative imaging, adjuvant radiation therapy, stereotactic radiosurgery, or chemotherapy was not initiated. The patient was managed with radiographic surveillance, with a plan for repeat MRI at one year to continue assessing long-term outcomes. A summary of the patient’s clinical timeline is provided in Table 1, and a comparison of selected low-grade posterior fossa gangliogliomas (GGs) with the case presented is provided in Table 2. 

**Table 1:** Timeline of clinical events.

Time point	Event
Day 0	Motor Vehicle Accident: screening head CT revealed incidental calcified midline cerebellar mass
Day 2	MRI brain with and without contrast demonstrated enhancing midline inferior cerebellar mass
Six weeks	Repeat MRI showed mild interval craniocaudal growth and partial fourth ventricle compression without hydrocephalus
Ten weeks/one month later	Midline suboccipital craniotomy and subtotal resection performed
Postoperative day 1	CT showed resection of central component with residual lateral calcification
Two months postoperative	MRI showed stable postoperative changes and residual tumor without hydrocephalus
Five months postoperative	Patient neurologically well with stable imaging; surveillance continued

**Table 2:** Selected reported cases of low-grade cerebellar and posterior fossa ganglioglioma compared with the present case. GG, ganglioglioma; ICP, intracranial pressure; MVA, motor vehicle accident.

Study/reference	Age/sex	Presenting symptoms	Tumor location	Key imaging findings	Treatment/Extent of resection	Follow-up/outcome
Handa et al. (1994) [5]	53 yr/M	Cerebellar symptoms (oldest reported cerebellar GG at time)	Cerebellum	Not specified (pre-modern MRI protocols)	Gross total resection	Symptom-free at 9 months postop
Safavi-Abbasi et al. (2007) [15]	29 yr/M	11-year history of intermittent headaches	Right cerebellar hemisphere with supratentorial extension to superior colliculi and pineal recess	CT initially negative; MRI: space-occupying lesion in right cerebellar hemisphere with supratentorial extension	Subtotal resection	Marked clinical improvement at 24 months; significant reduction in headache intensity/frequency
Bram et al. (2018) [11]	20 mo/M	Syncope, emesis (no seizures, no hydrocephalus, no elevated ICP)	Cerebellum	No radiographic hydrocephalus; no signs of elevated ICP	Gross total resection	Full resolution of preoperative symptoms; no new neurological deficits
Agrawal et al. (2018) [16]	17 yr/F	Cerebellar seizures	Cerebellum	Not specified (first cerebellar pigmented GG reported)	Tumor excision	Seizure resolution after excision
Tuan & Duc (2021) [7]	11 yr/M	Headache, epilepsy	Right cerebellar hemisphere (flattening fourth ventricle)	MRI: giant cyst with enhancing mural nodule in right cerebellar hemisphere; mimicked pilocytic astrocytoma	Gross total resection	Postoperative recovery; no recurrence reported
Komboz et al. (2024) [14]	56 yr/M	Asymptomatic (incidental finding during lung cancer workup)	Left lobulus semilunaris superior cerebelli	MRI: space-occupying lesion initially suspected to be cerebellar metastasis	Resection (to establish diagnosis)	No recurrence reported
Qin et al. (2014) [17]	35 yr/F	Headache, vertigo, ataxia, saccadic dysfunction, dysarthria, dysmetria (waxing-waning course over several years)	Pons and cerebellum (multiple lesions)	MRI: multiple lesions in pons and cerebellum with cystic changes; size fluctuation over years; mimicked inflammatory/demyelinating process	Biopsy only (3 biopsies required for diagnosis)	Progressive neurological deterioration over years prior to diagnosis
Bills & Hanieh (1991) [18]	Infant	Hemifacial spasm	Fourth ventricle	Not specified (fourth ventricular mass)	Surgical resection	Hemifacial spasm relieved by surgery
Present case	32 yr/ M	Incidental discovery following MVA	Cerebellum	Midline cerebellar mass with punctate calcifications	Subtotal resection	Symptom-free at 5 months post

## Discussion

GGs are rare mixed neuronal and glial tumors that represent <1% of CNS neoplasms [5]. They comprise a varied composition of neuronal and glial cells at differing degrees of differentiation, but it is typically the grade of the glial element that predicts biological behavior [7,10,11]. They are typically considered within the WHO grade I or II classification, which accounts for 90%-98% of cases, with rare cases of anaplastic GG [11]. Malignant degeneration of pre-existing WHO grade I GG into the anaplastic variety is also infrequent, with estimated transformation rates between 0.6%-14.5%, possibly secondary to long-standing residual tumor or radiation treatment [1,15,19].

The preponderance of cases arises in younger patients, and the large majority are located supratentorially, most commonly in the temporal lobes [1,5,7]. They may occur anywhere throughout the CNS, with infratentorial GG representing an exceedingly uncommon entity. Of those, the majority involve the brainstem, with purely cerebellar lesions comprising a smaller proportion [11]. The most common overall clinical manifestation is intractable seizures, secondary to temporal lobe localization. When they arise within the cerebellum, patients often present with symptoms of slowly progressing hydrocephalus due to compression of the fourth ventricle, such as headache, ataxia, nausea, myoclonus, and variable cranial nerve deficits [4,6,7,11]. Most patients have a protracted length from symptom onset until diagnosis, and peak incidence is between 10 and 20 years [4,10]. In contrast, our patient was diagnosed incidentally at greater than 30 years old, with only the eventual development of mild headaches.

There are wide variations in radiological findings from series to series, but in general, roughly half have cysts, calcifications, and/or enhancement [10]. Although a broad radiographic differential exists for enhancing posterior fossa lesions, the present case is specifically contextualized through comparison with selected published low-grade posterior fossa ganglioglioma cases in Table 2. It is classically described as a cyst with an enhancing mural nodule, but this appearance is not specific to GG [7]. The calcification pattern may be unusual and can be confused for vessels or vascular malformations [4]. In the present case, an internal susceptibility signal was identified on SWAN imaging; however, this finding can be difficult to distinguish from calcification, likely accounting for the concern for a vascular lesion and the subsequent negative CTA [20]. Additionally, these tumors typically have no surrounding edema and minimal mass effect [5]. Overall, owing to inconsistent and variable radiological findings, this tumor is seldom suspected based on imaging findings alone [4].

Optimal therapy for these lesions consists of gross total resection and is associated with disease control in over 95% of patients [1]. However, as in our patient, tumor extension and risk of significant neurological deficits with complete resection may preclude gross total resection. In those cases, subtotal resection, as undertaken in our patient, can adequately control tumor progression, with an approximate 15% long-term recurrence rate reported for all GGs [1]. However, infratentorial GGs may have a more benign clinical course, with subtotal resection achieving a comparable survival rate to gross total resection in supratentorial GGs [11]. A key limitation of this report is the limited duration of follow-up, as long-term surveillance was restricted to five months due to missed follow-up appointments. This limits our ability to draw definitive conclusions regarding long-term tumor control or recurrence risk after subtotal resection.

Radiation treatment remains contentious and is not routinely recommended for low-grade GG because of the typically indolent clinical course, potential deleterious effects of radiation, possible association with malignant degeneration, and the generally benign clinical course of cerebellar GG [1,7,11]. However, stereotactic radiosurgery may be considered in selected cases of residual, recurrent, progressive, or surgically inaccessible disease. In an international multicenter study, Mantziaris et al. reported a five-year progression-free survival rate of 85.6% after stereotactic radiosurgery for gangliogliomas [21]. Therefore, while stereotactic radiosurgery (SRS) was not used in this case, it remains a potential future consideration if the residual tumor demonstrates progression.

## Conclusions

Cerebellar gangliogliomas are rare infratentorial tumors that may present with nonspecific clinical and imaging findings, making preoperative diagnosis challenging. This case highlights that cerebellar ganglioglioma may be initially discovered incidentally in adults, despite the more typical association of posterior fossa tumors with headache, ataxia, nausea, vomiting, or hydrocephalus. Although the lesion was asymptomatic at discovery, interval growth and subsequent headaches prompted surgical intervention for tissue diagnosis and maximal safe resection. Imaging may also be misleading, as calcification and SWAN susceptibility signal can mimic vascular or hemorrhagic posterior fossa lesions, and intraoperative frozen section may be nondiagnostic. Surgical resection remains the primary diagnostic and therapeutic approach, particularly when tissue diagnosis is needed for an enlarging or indeterminate lesion. When gross total resection is not safely feasible due to infiltrative involvement of critical posterior fossa structures, subtotal resection followed by close radiographic surveillance may be appropriate. Longer follow-up is needed to better characterize tumor control and recurrence risk in incompletely resected adult cerebellar gangliogliomas.
